# Mathematical Based Calculation of Drug Penetration Depth in Solid Tumors

**DOI:** 10.1155/2016/8437247

**Published:** 2016-06-08

**Authors:** Hamidreza Namazi, Vladimir V. Kulish, Albert Wong, Sina Nazeri

**Affiliations:** ^1^School of Mechanical and Aerospace Engineering, Nanyang Technological University, Singapore 639798; ^2^Department of Radiotherapy, Oncology and Palliative Care, Sarawak General Hospital, 93586 Kuching, Sarawak, Malaysia; ^3^Faculty of Cognitive Sciences and Human Development, Universiti Malaysia Sarawak, 94300 Kota Samarahan, Sarawak, Malaysia

## Abstract

Cancer is a class of diseases characterized by out-of-control cells' growth which affect cells and make them damaged. Many treatment options for cancer exist. Chemotherapy as an important treatment option is the use of drugs to treat cancer. The anticancer drug travels to the tumor and then diffuses in it through capillaries. The diffusion of drugs in the solid tumor is limited by penetration depth which is different in case of different drugs and cancers. The computation of this depth is important as it helps physicians to investigate about treatment of infected tissue. Although many efforts have been made on studying and measuring drug penetration depth, less works have been done on computing this length from a mathematical point of view. In this paper, first we propose phase lagging model for diffusion of drug in the tumor. Then, using this model on one side and considering the classic diffusion on the other side, we compute the drug penetration depth in the solid tumor. This computed value of drug penetration depth is corroborated by comparison with the values measured by experiments.

## 1. Introduction

**Figure 1:**
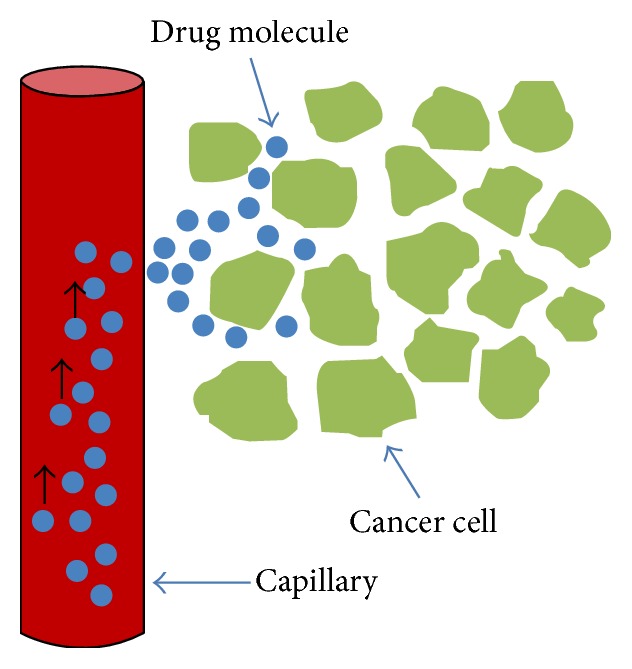
Drug delivery and diffusion in the cancer tumor.

Chemotherapy is an important option for cancer treatment which uses chemical substances (anticancer drug) to fight cancer. Considering application of drug through blood stream, drug travels to the cancer tumor and diffuses in it through capillaries as is shown in Figure 1.

Drug concentration in the tumor is dependent on drug production (supply, release, and activation), transport (diffusion and advection), and elimination (decay, deactivation, and cellular intake) [1]. These processes involve various biochemical, mechanical, and biophysical factors which make the process complex. Mathematical modeling provides a mean to better understand this complexity. Also mathematical modeling allows scientists to link the laboratory experiments with clinical applications by providing the means to extrapolate the in vivo results from mouse models to humans. There are valuable attempts in modeling of steps which affect drug concentrations, which have been reported in the literature. We can call the mathematical/computational models addressing drug vascular supply [2–6], drug release and activation [7–13], drug diffusive transport [14–24], drug advective transport [5, 25–27], and drug decay, deactivation, and cellular uptake [28–31].

In chemotherapy, it is also important that drug reaches the entire tumor, otherwise its effectiveness will be compromised [32]. So, in chemotherapy, a minimum effective concentration is required in all parts of the tumor for the effective treatment. One of the factors that come to account in discussion about drug concentration is drug penetration depth in the tumor. Penetration depth can be defined as the depth from a capillary at which the minimum concentration (required for cancer treatment) is achieved. Thus, studying and measuring the drug penetration depth in solid tumor are important issues in chemotherapy.

Beside numerous experimental studies which have been done on measuring the drug penetration depth in solid tumors [33–37], limited works on mathematical modeling have been reported in the literature. Using vascular images, Baish et al. designed a mathematical model that analyses drug diffusion in irregularly shaped domains based on maximum distance in the tissue from the nearest blood vessel (penetration depth) and a measure of the shape of the spaces between vessels. Their model predicted how new therapeutic agents alter the functional efficiency of the vasculature within the tumor tissue [38]. Welter and Rieger developed a vascular tumor growth model, including vessel cooption, regression, and angiogenesis, that extended by the interstitium, sources, and sinks for interstitial fluid flow (IFF). Using this model, they mathematically described the drug penetration depth (the length scale on which the solution decays around vessels). They found out that penetration depth for interstitial fluid pressure (IFP) across the tumor boundary has a variation in the radial profile. The experiments verified their modeling results [39].

The Thiele modulus, a dimensionless group, can be used to determine if a vesicle system is reaction or diffusion limited without solving any ordinary differential equation (ODE) or partial differential equation (PDE). Considering the characteristic, time for internalization and catabolism is faster than the time required for antibody to reach the most distant tumor tissue; Thurber et al. used Thiele modulus in order to develop a mathematical equation for drug concentration in tumors [40]. From this model, they computed the drug penetration depth in the tumor. The Thiele modulus was verified by experimental results where two datasets, which were not compounded by clearance effects, were chosen. Their model illustrates that, with the large resistance from the tumor capillary, antibodies may be more suitable for targeting micrometastases than vascularized tumors. Ackerman et al. rearranged the Thiele modulus found by Thurber et al. which yields an expression predicting the distance that a prevascular spheroidal metastasis will be penetrated by an antibody. The governed equation predicts that penetration distance will increase proportionally to antibody dose and inversely proportional to antigen expression level. Accordingly, a 10-fold decrease in antigen density is expected to yield a 10-fold increase in penetration distance. Similarly, the model also predicts that a 10-fold decrease in antigen density will negate the effect of a 10-fold decrease in antibody dose. They experimentally tested these predictions. Overall, the experimental results quantitatively validated the model of Thurber et al. and showed the importance of antigen-specific variables in tumor penetration [41].

In this research by using two types of diffusion equations, firstly we model the drug concentration in the solid tumor, and then we compute the drug penetration depth from equality of these models, as both of them explain the same phenomenon. The computed value will be compared with the real experiments' results. The interesting point about this model is that it considers the time delay for the rise of diffusion in the tumor.

## 2. Method

### 2.1. Drug Concentration in the Tumor

In order to study the drug penetration in the tumor, first we need to investigate about drug concentration in the tumor versus time. As it was mentioned in the last section, many researchers have done extensive works in this area.

**Figure 2:**
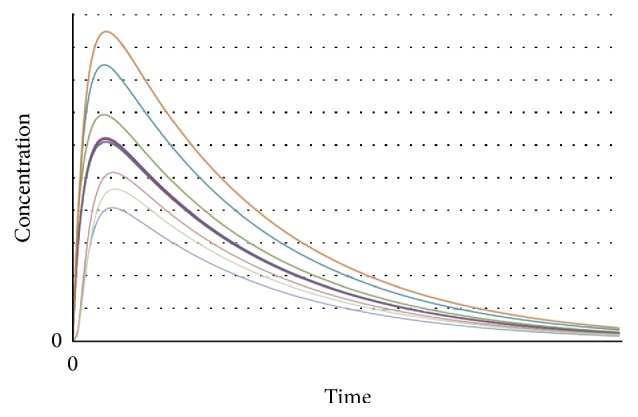
Anticancer drug concentration in the tumor versus time in case of different diffusivities.

Figure 2 shows the general behavior of variations of drug concentration in the solid tumor versus time in case of different diffusivities [42–44]. It is noteworthy that these concentration's values were determined with respect to the distance from vasculature. Also the dimensions of concentration and time can be varied in case of different works.

In Figure 2, lower diffusivity of tumor causes lower value for drug concentration. Also there is a general trend for variation of drug concentration in the tumor. As it can be seen in this figure, the drug concentration in the tumor increases with time to reach a maximum value. After reaching this maximum, concentration of drug decreases gradually till reaching a stable value. In fact, at first, the diffusion of drug in the tumor is high enough relative to the diffusion out of it to steadily increase the concentration over time. After the maximum concentration is reached, however, the diffusion-out is greater than the diffusion-in, and the concentration begins to decrease. It should be noted that based on the conditions (tumor diffusivity and structure, blood velocity, cellular uptake [29], etc.) the values of concentration, slope of curve, and the time periods in each step vary in case of different experiments. The general behavior seen in this plot can be seen in all investigations about anticancer drugs concentration in case of different types of cancers [42–44].

An interesting point in this plot is the time delay between the application of drug to the tumor and rise of its concentration to the maximum value. In fact always there is a lagging time that has not been considered in any studies yet. For instance in application of Verteporfin (anticancer drug) to patients and its activation by irradiation, Verteporfin produces highly reactive singlet oxygen which reacts within 40 nanoseconds and results in the destruction of membrane bound organelles within the cell. Calcium ions are released from organelles such as the mitochondria and the endoplasmic reticulum which induces cell apoptosis [45]. So there will be a time delay between the drug application and the rise of its concentration in tumor. This behavior should be seen in case of application of all drugs as diffusion phenomenon always has a time delay. Please note that, based on the conditions, the lagging time can be so small but not negligible.

Based on diffusion phenomenon and considering this time delay, in the next section we will develop our model which considers both aspects.

### 2.2. Phase Lagging Model of Drug Diffusion

Here, we model and analyze the drug diffusion in the solid tumor from a mathematical point of view using phase lagging equations. The steps to generate the model are described here. Please note that in this modeling we consider and analyze the drug concentration in the tumor only due to drug diffusion.

In our modeling, we introduce a time lag term that takes care of the lag between the arrival of drug to the cancer tumor and rise of its concentration in the tumor. Considering the diffusion phenomenon, the constitutive relation to be coupled with the conservation equation is

(1) $$\begin{array}{c} \Psi \left(\;x,t+\tau \;\right)=-D\frac{\partial W}{\partial x}\left(\;x,t\;\right). \end{array}$$

In this equation ∂*W*/∂*x* is the direct result of application of the drug to the system. Also *τ* is the time lag that ensures that the paradox of instantaneous propagation does not become a factor.

By expanding the left side of (1) with Taylor's series,

(2) $$\begin{array}{c} \Psi \left(\;x,t\;\right)+\tau \frac{\partial \Psi }{\partial t}\left(\;x,t\;\right)≅-D\frac{\partial W}{\partial x}\left(\;x,t\;\right). \end{array}$$

We derive (2) with respect to *x*:

(3) $$\begin{array}{c} \Psi_{x}+\tau \frac{\partial \Psi_{x}}{\partial x}≅-DW_{xx}. \end{array}$$

Ψ_*x*_ can be written from the following conservation equation:

(4) $$\begin{array}{c} \Psi_{x}=-W_{t}+f\left(\;x,t\;\right). \end{array}$$

In (4), *f*(*x*, *t*) is the source/sink function that can be related to drug decay, deactivation, cellular uptake, and so forth.

By substituting (4) into (3),

(5) $$\begin{array}{c} \tau \frac{\partial^{2}W}{\partial t^{2}}+\frac{\partial W}{\partial t}=D\frac{\partial^{2}W}{\partial x^{2}}+f\left(\;x,t\;\right)+\tau f_{t}\left(\;x,t\;\right). \end{array}$$

Considering

(6) $$\begin{array}{c} S\left(\;x,t\;\right)=f\left(\;x,t\;\right)+\tau f_{t}\left(\;x,t\;\right) \end{array}$$

as the general source/sink term, then

(7) $$\begin{array}{c} \tau \frac{\partial^{2}W}{\partial t^{2}}+\frac{\partial W}{\partial t}=D\frac{\partial^{2}W}{\partial x^{2}}+S\left(\;x,t\;\right). \end{array}$$

In (7), *τ* = *D*/*c* ^2^. In this research, *c* is the speed of the drug propagation through the solid tumor. The diffusivity term, *D*, is related to the resistance of the tumor to the drug; this is the property of the tumor tissue which will dampen the drug as it travels over the tissue.

The source/sink term, *S*(*x*, *t*), is not considered here as the only effect is related to drug diffusion in the tumor.

The initial conditions are defined as *W*_0_ = 0 mol/m^3^ and ∂*W*/∂*t* = 0 at *t* = 0.

In order to solve (7), we introduce three new variables:

(8) −ξ=xD1/2,ϑ=tτ1/2,Λ=W−W0.

The term *ξ* is the new distance-like variable, *ϑ* is the new time-like variable, and Λ is the excess value of target parameter, which is drug concentration in this research. By changing the variables, (7) becomes

(9) $$\begin{array}{c} \frac{\partial^{2}\Lambda }{\partial \vartheta^{2}}+\tau^{-1/2}\frac{\partial \Lambda }{\partial \vartheta }=\frac{\partial^{2}\Lambda }{\partial \xi^{2}}. \end{array}$$

The new initial conditions are defined as Λ = 0 and ∂Λ/∂*ϑ* = 0 at *ϑ* = 0.

Applying Laplace Transform to (9),

(10) $$\begin{array}{c} \frac{d^{2}\tilde{\Lambda }}{d\xi^{2}}-s\left(\;s+\tau^{-1/2}\;\right)\tilde{\Lambda }=0. \end{array}$$

The general solution of (10) is

(11) Λ~ξ,s=B1sexp⁡−ξss+τ−1/21/2+B2sexp⁡ξss+τ−1/21/2.

In fact, the solution has to be bounded when *ξ* → *∞*, as we consider the solid tumor as the domain. This requires that *B*_2_(*s*) → 0. Denoting *B*_1_(*s*) ≡ *B*(*s*),

(12) $$\begin{array}{c} \tilde{\Lambda }\left(\;\xi ,s\;\right)=B\left(\;s\;\right)\exp\left\{\;-\xi {\left[\;s\left(\;s+\tau^{-1/2}\;\right)\;\right]}^{1/2}\;\right\}. \end{array}$$

From (12), *B*(*s*) is

(13) $$\begin{array}{c} B\left(\;s\;\right)=\left[\;\tilde{\Lambda }\left(\;\xi ,s\;\right)\;\right]\exp\left\{\;\xi {\left[\;s\left(\;s+\tau^{-1/2}\;\right)\;\right]}^{1/2}\;\right\}. \end{array}$$

In order to eliminate *B*(*s*), we differentiate (12) with respect to *ξ*:

(14) dΛ~dξ=−ss+τ−1/21/2Bs·exp⁡−ξss+τ−1/21/2=−ss+τ−1/2·Λ~ξ,s.

Thus,

(15) $$\begin{array}{c} -\tilde{\Lambda }\left(\;\xi ,s\;\right)={\left[\;s\left(\;s+\tau^{-1/2}\;\right)\;\right]}^{-1/2}\frac{d\tilde{\Lambda }}{d\xi }. \end{array}$$

By taking the inverse Laplace Transform of (15),

(16) $$\begin{array}{c} \Lambda =-\overset{\vartheta }{\underset{0}{\int }}\frac{\partial \Lambda }{\partial \xi }T_{0}\left(\;\frac{\vartheta -\xi }{2\sqrt{\tau }}\;\right)\exp\left(\;-\frac{\vartheta -\xi }{2\sqrt{\tau }}\;\right)d\xi . \end{array}$$

And then restoration of the original terms is as follows:

(17) Wx,t=W0−Dτ1/2∫0t∂W∂xT0t−t∗2τexp⁡−t−t∗2τdt∗.

Equation (17) provides the relationship for computation of drug concentration.

The term ∂*W*/∂*x* is related to the flux as ∂*W*/∂*x* = −(1/*D*)*∅*, where *∅* is the flux. Applying this relation to (17),

(18) Wx,t=W0+1Dτ1/2∫0t∅T0t−t∗2τexp⁡−t−t∗2τdt∗.

In [14] we have showed that *∅* can be modeled as a Gaussian pulse:

(19) $$\begin{array}{c} \emptyset =A\exp\left[\;-\frac{{\left(t-t_{max}\right)}^{2}}{\sigma^{2}}\;\right], \end{array}$$

where *A* is the maximum flux, *t* is the total time, *t*_max_ is the time of maximum flux, and *σ* is the standard deviation of flux.

By substituting (19) into (18),

(20) Wx,t=W0+1Dτ1/2∫0tAexp⁡−t−tmax2δ2·T0t−t∗2τexp⁡−t−t∗2τdt∗.

**Figure 3:**
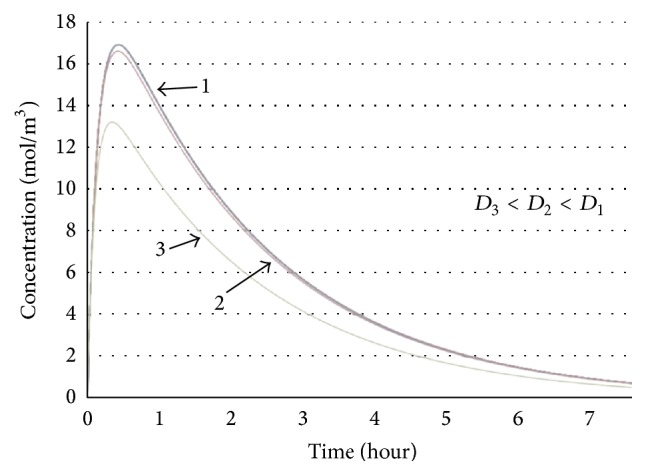
The solution of (20) in case of different diffusion coefficients and time lags. Drug concentration versus time.

Equation (20) is solved for three values of diffusion coefficient and lagging time and plotted in Figure 3. It shows drug concentration versus time in the solid tumor.

As it can be seen in Figure 3, the drug concentration in all cases rises to the maximum points. The time delay in all cases is clear in this figure. After reaching the maximum amount, the drug concentration decreases gradually till reaching a stable value. As it is clear in the plot, bigger values of diffusion coefficient cause higher peak in the concentration plot. This behavior mimics the trend seen in Figure 2, where at first the diffusion of drug in the tumor is high enough relative to the diffusion out of it to steadily increase the concentration over time. Then, after the maximum point, the diffusion-out is greater than the diffusion-in, and the concentration begins to decrease. So phase lagging model defines the drug concentration in tumors well and can be used for our further investigations.

It should be noted that based on the value of parameters (diffusion coefficient, reaction time, and drug specifications), the values of concentration, slope of curve, and the time periods in each step vary in case of different experiments.

## 3. Result

In the last section, we developed (20) which indicates the relationship between the drug concentration and its spatial derivative within the tumor.

From another view, writing the behavior of drug propagation in the solid tumor by the classical model of diffusion,

(21) $$\begin{array}{c} W\left(\;x,t\;\right)=W_{0}-\sqrt{D_{eff}}\overset{t}{\underset{0}{\int }}\frac{\partial W}{\partial x}\frac{1}{\sqrt{\pi \left(t-t^{*}\right)}}dt^{*}. \end{array}$$

In (21), the effective diffusion coefficient, *D*_eff_, must be a function of time. In order to find *D*_eff_, (17) and (21) are combined:

(22) ∫0t∂W∂xDτT0t−t∗2τexp⁡−t−t∗2τ−Deff1πt−t∗dt∗=0.

Considering that (22) is valid for any *t* > 0 and dividing both sides of this equation by ∂*W*/∂*x* ≠ 0,

(23) $$\begin{array}{c} \sqrt{\frac{D}{\tau }}T_{0}\left(\;\frac{t}{2\tau }\;\right)\exp\left(\;-\frac{t}{2\tau }\;\right)-\sqrt{D_{eff}}\frac{1}{\sqrt{\pi t}}=0; \end{array}$$

hence,

(24) $$\begin{array}{c} D_{eff}=D\frac{\pi t}{\tau }{\left[\;T_{0}\left(\;\frac{t}{2\tau }\;\right)e^{-t/2\tau }\;\right]}^{2}. \end{array}$$

By replacing the temporal variable (*t*) by speed of drug propagation (*c*) and spatial variable (*x*) through

(25) $$\begin{array}{c} t=\frac{x}{c} \end{array}$$

and considering *δ* = *cτ*, we can write *D*_eff_ as a function of *x*:

(26) $$\begin{array}{c} D_{eff}=D\frac{\pi x}{\delta }{\left[\;T_{0}\left(\;\frac{x}{2\delta }\;\right)e^{-x/2\delta }\;\right]}^{2}. \end{array}$$

Here we introduce two dimensionless variables:

(27) D−=DeffD,η=xδ.

Using these variables, we rewrite (26) in a dimensionless form:

(28) $$\begin{array}{c} \overset{-}{D}=\pi \eta {\left[\;T_{0}\left(\;\frac{\eta }{2}\;\right)e^{-\eta /2}\;\right]}^{2}. \end{array}$$

Equation (28) provides the relationship between the diffusion coefficient and the spatial variable in dimensionless forms.

**Figure 4:**
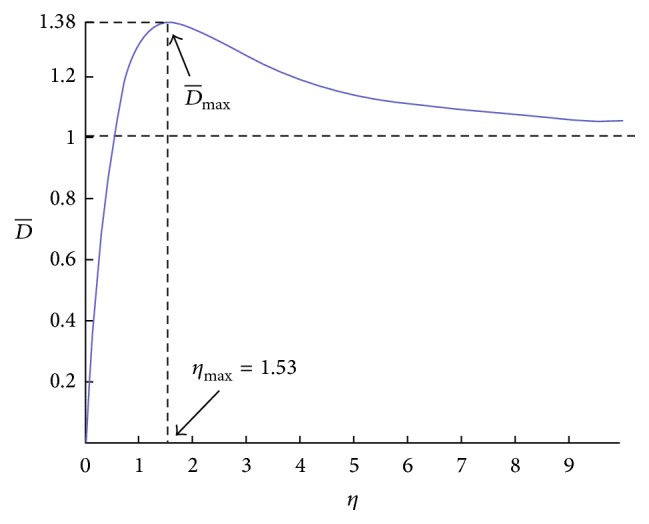
The variation of dimensionless diffusivity with the dimensionless spatial variable.

The variation of dimensionless diffusivity with the dimensionless spatial variable is shown in Figure 4.

Figure 4 shows that dimensionless diffusivity increases as *η* increases to reach its maximum value at *η*_max_. Based on (28), maximum dimensionless diffusivity at *η*_max_ = 1.53 has the following value:

(29) $$\begin{array}{c} {\overset{-}{D}}_{max}=1.38. \end{array}$$

Beyond ${\overset{-}{D}}_{max}$, the value of $\overset{-}{D}$ starts to decrease, and after some time interval, its value reaches a constant value. So the spatial position of ${\overset{-}{D}}_{max}$, which is *η*_max_ = 1.53, becomes a critical point.

Recalling *η* = *x*/*δ*, we name

(30) $$\begin{array}{c} x=\eta_{max}\delta \end{array}$$

“drug penetration depth.” In fact, anticancer drug can penetrate in the solid tumor at this depth. We claim that this drug penetration depth should be the same as drug penetration depth measured from experiments. Computing the drug penetration depth using (30) verifies this issue.

**Figure 5:**
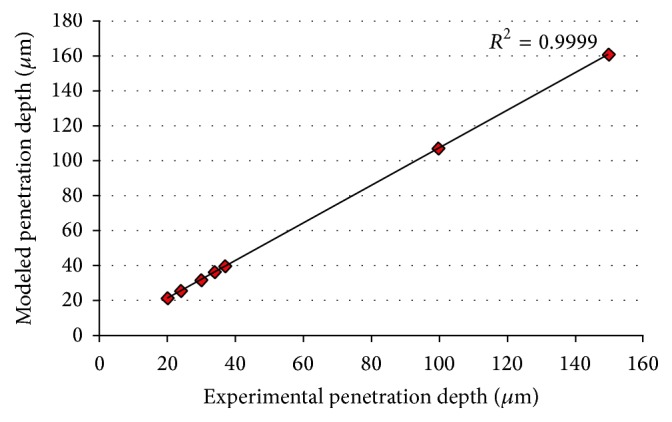
Comparison between the experimental and modeled values of drug penetration depth in case of different drugs.

Based on (30), here we computed the penetration depth in case of different drugs (look at Table 1) and compared the results with the experimental results (in case of animals or humans, in different types of cancer) done by other researchers [46–52] (look at Figure 5). Please note that the required values of parameters for our calculations in each case were brought from the literature. It is noteworthy that the value of reaction time in some cases governed through its formulation with *c* and *D*. Also (look at Table 1) it is noteworthy that the reason of having the same drug but different experimental and modeled penetration depth values in some cases is due to the application of drug in case of different types of cancer or the condition of application (for instance animal versus humans). The value of *R*-squared (*R* ^2^ = 0.9999) in the scatter plot shows very good fit. We also computed root mean squared error (RMSE). The computed value of 5.49 *μ*m for root mean squared error stands for acceptable accuracy of our model (attention to the range of variation of experimental penetration depth values) for prediction of penetration depth in case of different drugs.

Also please note that in all cases the computed value is bigger than the experimental value. It is due to the fact that modeling conditions differ from real conditions and all these conditions will cause less penetration of drug in the tumor. For instance in the real experiment there are some decays of drugs that have not been considered in the modeling steps.

In this research, we successfully developed a model for computation of drug penetration depth in solid tumors. Based on the required penetration depth in case of each tumor, the values of parameters from (30) can be advised. So the proper drug(s) with the required dosage can be applied based on the modeling outcome and considering the patient's general health condition. So this method can speed up clinical practice for cancer patients and drug, design development, and therapy for cancer drugs.

## 4. Discussion

In this paper, first we proposed the phase lagging model for drug diffusion in the solid tumors. This model provides a relation between the drug concentration in the solid tumor and its spatial derivative at any given moment of time and location. Using this model on one side and classical diffusion model on the other side, we considered the equality of these models within a one-dimensional domain (solid tumor). As a result, dependence of the diffusivity in a dimensionless form on the spatial domain was studied, and a characteristic length was found that has same characteristics as drug penetration depth in the solid tumor. In fact, beyond this length, effective diffusion starts to decrease, and thus the drug starts to disappear. Comparison of the computed penetration depth using this model with the drug penetration depth measured in the real experiments verified the significance of our claim. The efficiency of this method enables fast, accurate, and comprehensive computation of drug penetration depth in solid tumors. This technique can be used in case of different drugs applied in treatment of different types of cancer.

It is noteworthy that our mathematical model can be developed to be more precise by considering some phenomena and factors (such as some environmental factors) that have dominant effect on drug diffusion and penetration in the solid tumor. In this way, the error caused in our prediction will be reduced.

Similar to most of the mathematical models in biology and medicine, the mathematical model proposed in this research is based on some experimental data. The advantage of using this model over direct measurement of drug diffusion and penetration depth is the abilities that this model provides for us. For instance, this model is well suited for testing combinations of multiple parameters that can be varied simultaneously in a controlled manner and over a wide range of values. Such a broad screening of drug or tissue conditions is a hard and expensive job in laboratory experiments, but it is relatively easy and cheap in computer simulations. These theoretical screenings can help to determine the properties of therapeutic compounds which are optimal for their efficient interstitial transport or make decisions regarding the most effective drug and its application protocol.

In general, the mathematical models that are properly integrated with experimental data, such that both models and laboratory experiments inform each other, can provide tools for interpreting data, evaluating the most important parameters for designing new experiments, and developing strategies to improve tumor treatment.

## Figures and Tables

**Table 1:** The experimental and modeled values of drug penetration depth.

Drug	Experimental penetration depth (*μ*m)	Modeled penetration depth (*μ*m)
Doxorubicin (Dox-LTSL)	78	83.2
Doxorubicin (Doxil liposomes)	34	36.4
Cisplatin	20	21.3
Docetaxel	100	107.8
Paclitaxel	100	105.9
Fluorouracil	24	25.5
Mitoxantrone	37	39.4
Paclitaxel	30	31.9
Herceptin	150	160.7
Topotecan	100	106.8
